# Effects of Alpha-2 Adrenergic Agonist Mafedine on Brain Electrical Activity in Rats after Traumatic Brain Injury

**DOI:** 10.3390/brainsci11080981

**Published:** 2021-07-25

**Authors:** Yuriy I. Sysoev, Veronika A. Prikhodko, Roman T. Chernyakov, Ruslan D. Idiyatullin, Pavel E. Musienko, Sergey V. Okovityi

**Affiliations:** 1 Department of Pharmacology and Clinical Pharmacology, Saint Petersburg State Chemical and Pharmaceutical University, 197022 Saint Petersburg, Russia; veronika.prihodko@pharminnotech.com (V.A.P.); rom4ik19.06.2001@gmail.com (R.T.C.); rusidi100@gmail.com (R.D.I.); sergey.okovity@pharminnotech.com (S.V.O.); 2 Laboratory of Neuroprosthetics, Institute of Translational Biomedicine, Saint Petersburg State University, 199034 Saint Petersburg, Russia; pol-spb@mail.ru; 3 Pavlov Institute of Physiology RAS, 199034 Saint Petersburg, Russia; 4 N.P. Bechtereva Institute of the Human Brain of the Russian Academy of Sciences, 197376 Saint Petersburg, Russia; 5 Neuroscience Program, Sirius National Technical University, 354340 Sochi, Russia

**Keywords:** traumatic brain injury, neuroprotection, electrocorticography, rat, mafedine, dexmedetomidine

## Abstract

The search for and development of new neuroprotective (or cerebroprotective) drugs, as well as suitable methods for their preclinical efficacy evaluation, are priorities for current biomedical research. Alpha-2 adrenergic agonists, such as mafedine and dexmedetomidine, are a highly appealing group of drugs capable of reducing neurological deficits which result from brain trauma and vascular events in both experimental animals and human patients. Thus, our aim was to assess the effects of mafedine and dexmedetomidine on the brain’s electrical activity in a controlled cortical-impact model of traumatic brain injury (TBI) in rats. The functional status of the animals was assessed by electrocorticography (ECoG), using ECoG electrodes which were chronically implanted in different cortical regions. The administration of intraperitoneal mafedine sodium at 2.5 mg∙kg^−1^ at 1 h after TBI induction, and daily for the following 6 days, restored interhemispheric connectivity in remote brain regions and intrahemispheric connections within the unaffected hemisphere at post-TBI day 7. Animals that had received mafedine sodium also demonstrated an improvement in cortical responses to photic and somatosensory stimulation. Dexmedetomidine at 25 μg∙kg^−1^ did not affect the brain’s electrical activity in brain-injured rats. Our results confirm the previously described neuroprotective effects of mafedine sodium and suggest that ECoG registration and analysis are a viable method evaluating drug efficacy in experimental animal models of TBI.

## 1. Introduction

Traumatic brain injury (TBI) is a leading cause of disability and long-term loss of working capacity in working-age and socially active adults worldwide [1]. To date, a number of neuroprotective (or cerebroprotective) drugs have been proposed to attenuate neurological deficits resulting from brain trauma, but their clinical effectiveness remains mostly insufficient [2,3]. A major problem for the development of new drugs aimed at improving neuronal survival in traumatic and/or ischemic conditions is the limited translational potential of experimental animal studies. To address this problem, novel approaches to preclinical research of neuroprotective drug candidates should be sought and validated. Today, behavioral and functional testing, biochemical, and immunohistochemical methods are the conventional standards for evaluating pharmacological agents aimed at treating neurological impairments which result from brain trauma and other central nervous-system disorders. Other neuroimaging techniques such as magnetic resonance, computed, and positron emission tomography, are much less commonly used, which is explained by their expensiveness and the high spatial resolutions that are required for small animals.

Registration and analysis of the brain’s electrical activity (electroencephalography, EEG; electrocorticography, ECoG) are also used in experimental studies of rodents [4,5]. In several pilot studies, we examined and described typical alterations of brain electrical activity observed in rats subjected to penetrating TBI [6,7,8,9]. Despite the fact that a number of labs successfully apply EEG/ECoG techniques to study epileptiform activity in traumatized rats [10,11], the overall number of works currently employing these techniques to study pharmacological neuroprotection remains fairly low [12,13]. However, using EEG for this purpose has several definite advantages. Besides assessing the functional status of specific brain regions by means of amplitude and spectral analyses, it permits the examination of the intra- and interhemispheric connectivity using cross-correlation and coherence analyses. Evoked potential registration can be used to assess the function of a sensory system, including visual, auditory, somatosensory, and other systems. Moreover, brain electrical activity can be repeatedly measured in order to continuously monitor the functional status of the subjects. 

Among the many drugs aimed to reduce the severity of neurological symptoms, alpha-2 adrenergic receptor agonists represent a prominent group. The most convincing evidence for the neuroprotective activity of one of its members, dexmedetomidine, has been provided by a meta-analysis of 9 randomized placebo-controlled studies in 879 ischemic stroke patients [14]. The drug was shown to suppress the release of proinflammatory cytokines and neuroendocrine hormones, maintain adequate cerebral blood flow, and mitigate brain damage. Neuroprotective properties of another alpha-2 agonist, mafedine (6-oxo-1-phenyl-2-(phenylamino)-1,6-dihydropyrimidin-4-ol, in form of sodium salt) have been described in a rat model of TBI. Chronic administration of this compound-reduced brain damage and inhibited inflammation within the affected area, which was accompanied by an overall improvement in the neurological function of the brain-injured animals [15].

To confirm our previous results and further describe the neuroprotective properties of this drug, we have conducted an experimental study aimed at comparing the effects of mafedine sodium on the brain’s electrical activity to those of dexmedetomidine in a rat model of TBI.

## 2. Materials and methods

Animal experiments were carried out in compliance with the principles of the Basel Declaration, the Order of the Ministry of Health of the Russian Federation No. 199n (1 April 2016) “On the approval of the Rules of Good Laboratory Practice”, and the recommendations of the Bioethics Committee of the St. Petersburg State Chemical and Pharmaceutical University of the Ministry of Health of the Russian Federation. A total number of 32 white outbred male rats weighing 250–300 g were purchased from the Rappolovo laboratory animal supplier (Leningrad Oblast). All animals were received in a single shipment, quarantined for 2 weeks, then housed in a standard animal facility with free access to normal chow and drinking water.

Each animal was given an identification number and randomized using a random number method into one of the four groups: (1) presumably healthy controls; (2) TBI; (3) TBI + mafedine sodium, 2.5 mg∙kg^−1^ b.w.; and (4) TBI + dexmedetomidine, 25 μg∙kg^−1^ b.w. All experimental groups consisted of 8 animals. The dose of mafedine was chosen based on our previous results [15], and that of dexmedetomidine was chosen according to literature data [16,17,18].

Corticographic electrodes were made of nichrome wire with a diameter of 0.5 mm (active and reference electrodes) or 0.16 mm (ground electrode). The electrodes were insulated with heat-shrink tubing, leaving a non-insulated conductive tip of ~1 mm in length. All electrodes were assembled in a single-row, 8-pin, 2.54 mm pitch, straight female header connector.

**Figure 1:**
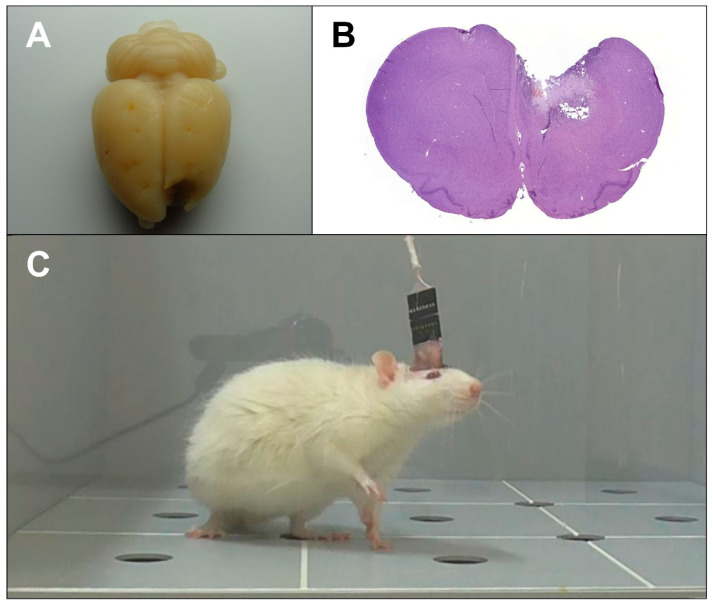
(**A**) macroscopic view of an injured rat brain at post-TBI day 7. Electrode and fastening screw marks are visible on the brain surface; (**B**) brain section at the level of electrodes FP1 and FP2 obtained from the same rat, stained with haematoxylin-eosin. A deep lesion involving both the cortex and underlying structures is visible in the left hemisphere; (**C**) the rat with implanted electrodes.

Prior to surgery, the animals were anesthetized with chloral hydrate (400 mg∙kg^−1^ b.w.), and carbomer-based eye gel (Ophthagel^®^, Santen OY, Finland) was applied to prevent corneal drying. After shaving and skin disinfection, a mid-line incision was made from the base of the skull to the frontal bone. To expose the cranial surface, the muscles, fascia, and periosteum were retracted. Thermocoagulation was provided as necessary at all times. Following cranial surface preparation, burr holes up to 1 mm deep were drilled for the electrodes and fastening screws. Intermittent drilling was performed in order to prevent excessive frictional heat generation and the subsequent thermal brain damage. Presumably healthy animals were then implanted with corticographic electrodes fastened with screws, while all other rats were subjected to TBI (Figure 1A,B) using the method described earlier [19].

To model TBI, a craniectomy was performed in the left-frontal region above the sensorimotor cortex, centered at 2.0 mm rostral and 1.5 mm left-lateral from the bregma. A steel-guide tube, carrying a piston with a diameter of 3 mm and a stroke length of 4 mm, was positioned over the cranial opening. To produce a brain injury, the piston was actuated by a 50 g weight sliding down the guide tube from a height of 10 cm. The removed bone flap was then placed back, and the electrodes were implanted and fastened with screws.

The electrode coordinates were determined using the Paxinos & Watson stereotaxic rat brain atlas [20]. Electrodes FP1 and FP2 were placed in the secondary motor cortex area (AP = +2.0, ML = 1.5, DV = 1.0), C3 and C4, the hindlimb primary motor cortex area (AP = −1.0, ML = 2.0, DV = 1.0), and O1 and O2, the primary somatosensory cortex area above the hippocampus (AP = −4.0, ML = 2.0, DV = 1.0). The reference electrode was implanted in the nasal bone, and the ground electrode was placed under the skin in the neck region. The implants were additionally fixated with Villacryl C dental acrylic resin (Zhermack, Italy) (Figure 1C). The incision was closed, and the suture and the surrounding area were disinfected with iodine solution. The drugs were administered at 1 h after TBI induction and then daily for the next 6 d according to the schedule (Table 1).

Following the surgery, the rats were kept in individual cages with free access to standard chow and drinking water until the end of the experiments. Each animal’s condition was checked at recovery from anesthesia and then monitored twice a day, in the morning and in the evening. Suture disinfection with iodine solution was provided as necessary. In order to prevent dehydration, saline was administered subcutaneously for the first 3 days after the surgery. We deliberately avoided the use of antibiotics, analgesics, and anti-inflammatory drugs because the majority of them are known to affect, at least to some extent, the course of pathological changes observed in TBI, which could distort the results of the study [21,22].

Cortical-electrical activity was recorded on post-TBI days 3 and 7 using an 8-channel Neuron-Spectrum-1 EEG system (Neurosoft, Russia) at a 0.5–35 Hz bandwidth and a 500 Hz sampling rate. The two time points were chosen in order to assess the brain function of the injured animals in the acute (day 3) and subacute (day 7) phases of TBI [8]. Both home-cage spontaneous cortical-electrical activity and evoked potentials were strictly recorded according to the schedule (Table 1).

Corticogram fragments of up to 5 min long which corresponded to awake, resting state with no locomotion, exploratory and/or grooming behavior were analyzed by Neuron-Spectrum.NETω software (Neurosoft, Russia). For amplitude analysis, an overall mean of the wave amplitudes were calculated. Spectral analysis included the calculation of mean wave amplitudes and rhythm indices for each of the δ (0.5–4.0 Hz), θ (4.0–8.0 Hz), α (8.0–14.0 Hz), and β frequency bands (low-frequency, LF—14.0–20.0 Hz, and high-frequency, HF—20.0–35.0 Гц). Rhythm indices were calculated as total duration percentages of signals registered in the δ, θ, α, and β frequency bands. Cross-correlation analysis included the calculation of cross-correlation coefficients (C_Cr_) for the following electrode pairs: FP1-FP2, C3-C4, and O1-O2 (horizontal), and FP1-C3, FP2-C4, C3-O1, and C4-O2 (vertical). The analyzed epoch length was set to 5 s.

Visual-evoked potentials (VEPs) were recorded on post-TBI days 3 and 7. Flash photostimulation was performed using a PhS-1 white-light diode photic stimulator (Neurosoft, Russia) over 30 s periods at a stimulation frequency of 3 Hz and a stimulus duration of 50 ms. These stimulation parameters were found to be optimal for evoking consistent cortical responses in preliminary validation studies. VEP curves were analyzed using Neuron-Spectrum-DVP.NET software (Neurosoft, Russia). For all channels, P2 peak amplitude was measured since it has been previously shown to undergo the most specific changes in TBI in rats [9].

Somatosensory-evoked potentials (SSEPs) were recorded at post-TBI day 7, immediately after spontaneous cortical activity and VEP registration. Prior to the procedure, the animals were anesthetized with chloral hydrate (400 mg·kg^−1^ b.w. i.p.). Electrical stimulation (2 mA, 0.1 ms square-wave pulse stimuli at 1 Hz rate) was applied to *n. sciaticus* using a Neuro-MEP electrical stimulator (Neurosoft, Ivanovo, Russia), alternating between right and left extremities. The stimulation parameters adequate for evoking a strong response were chosen according to the literature [23] with slight modifications based on our preliminary results [6]. Cortical responses to sciatic nerve stimulation were recorded in the C3 and C4 regions. SSEP curves were also analyzed using Neuron-Spectrum-DVP.NET software (Neurosoft, Ivanovo, Russia). The N1, P2, N2, P3, and N3 peak latencies and amplitudes were measured. The analyzed epoch length was set to 350 ms, and individual responses were averaged in sets of 30.

Statistical analysis of the data was performed using GraphPad Prism 7.00 software (GraphPad Software Inc., San Diego, CA, USA). The data were tested for normality using the Shapiro-Wilk W-test. For normally distributed data, the significance of differences between group means was tested using one-way ANOVA followed by Dunnett’s multiple comparison *post hoc* test. Otherwise, the Kruskal-Wallis non-parametric test followed by Dunn’s *post hoc* test was used. The significance threshold was set at *p* < 0.05.

## 3. Results

Our experiments suggest that unilateral left-sided TBI causes distinct amplitude and spectral changes in the ECoG signal, disrupts inter- and intrahemispheric functional connectivity, and alters VEP and SSEP curve parameters in rats. Out of the 32 rats, 2 (1 in mafedine and 1 in dexmedetomidine group) died between post-surgery days 3 and 7 and thus could not be included in the last testing session.

**Figure 2:**
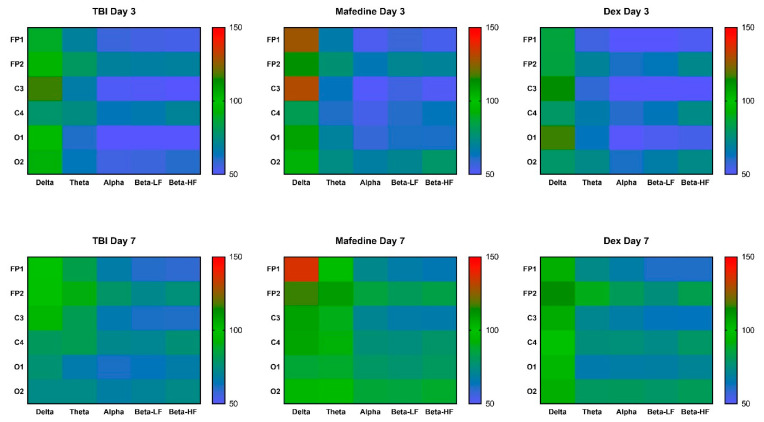
A heatmap of mean δ-, θ-, α-, LF and HF β-wave amplitudes in channels FP1, FP2, C3, C4, O1, and O2 at post-TBI days 3 and 7. Data were normalized to the respective healthy control values.

**Figure 3:**
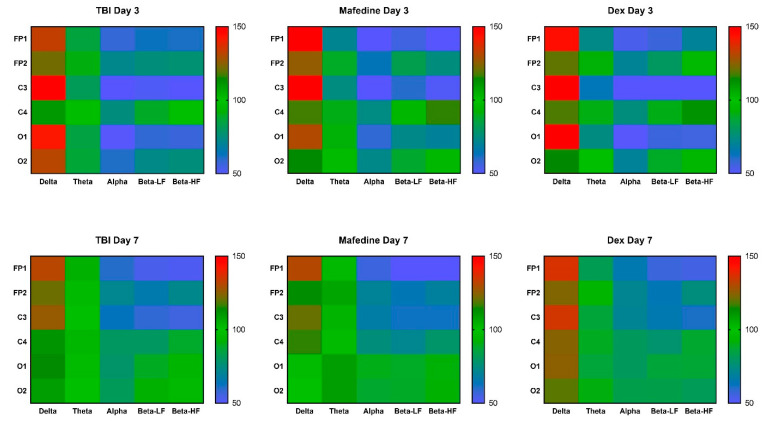
A heatmap of δ-, θ-, α-, LF and HF β-rhythm index values in channels FP1, FP2, C3, C4, O1, and O2 at post-TBI days 3 and 7. Data were normalized to the respective healthy control values.

Mean ECoG signal amplitudes in all the channels were significantly lower in TBI rats than in presumably healthy controls, at both post-TBI days 3 and 7 (*p* < 0.05, *p* < 0.01) (Table 2). Furthermore, all mean rhythm amplitudes, except δ-rhythm, decreased in TBI animals at both time points (*p* < 0.05, *p* < 0.01) (Figure 2). Rhythm-index analysis also revealed some very distinct changes: the power spectrum in the injured hemisphere (channels FP1, C3, and O1) was dominated by δ frequencies, while θ-, α-, LF and HF β-waves decreased (*p* < 0.05, *p* < 0.01). At the same time, the rhythm indices in the unaffected hemisphere did not significantly differ from control levels (Figure 3).

Neither mafedine nor dexmedetomidine significantly affected the amplitude and spectral parameters of the corticograms obtained from the brain-injured rats at post-TBI days 3 or 7. However, mafedine-treated animals showed a clear tendency towards an increase in mean signal amplitudes and mean amplitudes of all frequencies in occipital channels when compared to controls (Table 1, Figure 2). At post-TBI day 3, mafedine-treated rats also had higher mean δ-rhythm amplitudes at channels FP1 and C3 than controls, with a slight decrease by day 7 (Figure 2).

**Figure 4:**
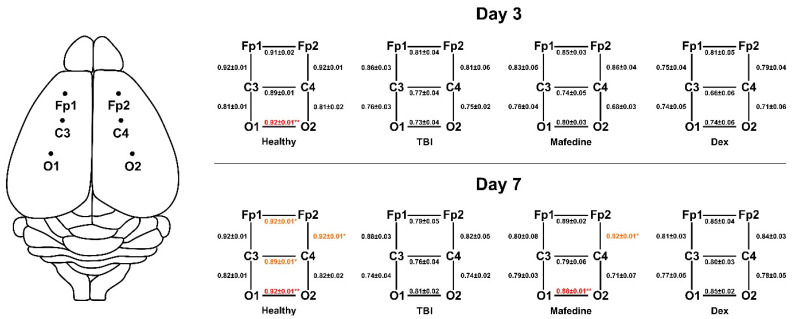
Ccr values for electrode pairs FP1-FP2, C3-C4, O1-O2, FP1-C3, FP2-C4, C3-O1, and C4-O2 at post-TBI days 3 and 7. Left: active electrode placement layout. Dex dexmedetomidine; * *p* < 0.05, ** *p* <0.01 vs. TBI.

Lower cross-correlations of all electrode pairs were another common feature seen in TBI rats, indicating an impairment of both inter- and intrahemispheric functional connections. The Ccr values significantly decreased (*p* < 0.05, *p* < 0.01) for electrode pair O1-O2 by post-TBI day 3, and pairs FP1-FP2, C3-C4, O1-O2, and FP2-FP4 by post-TBI day 7 (Figure 4). Mafedine administration increased Ccr values for electrode pairs FP2-C4 (*p* < 0.05) and O1-O2 (*p* < 0.01) compared to TBI group at post-TBI day 7 (Figure 4).

**Figure 5:**
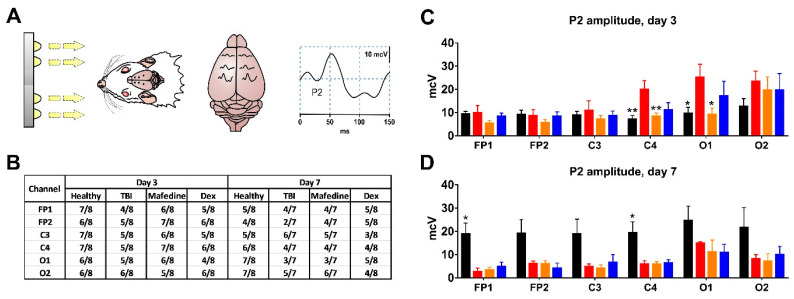
(**A**) a schematic representation of the VEP acquisition process. Right: an example of obtained VEP curve with a highlighted P2 peak; (**B**) P2 peak incidence in experimental animals at post-TBI days 3 and 7; (**C**,**D**) P2 peak amplitudes in channels FP1, FP2, C3, C4, O1, and O2 at post-TBI days 3 and 7. Black healthy controls, red TBI, orange TBI + mafedine, blue TBI + dexmedetomidine (Dex). Dex dexmedetomidine; * *p* < 0.05, ** *p* <0.01 vs. TBI.

TBI had no effect on the incidence of the P2 VEP peak at either post-TBI day 3 or 7 (Figure 5A,B). Rats subjected to TBI had significantly higher P2 peak amplitudes in channels C4 and O1 (*p* < 0.01 and *p* < 0.05, respectively) at post-TBI day 3 when compared with presumably healthy controls (Figure 5C). A similar change pattern was noted for channel O2, although the significance level was not reached. At post-TBI day 7, P2 peak amplitudes decreased in all channels (in channels FP1 and C4, significantly at *p* < 0.05) in comparison with controls. At post-TBI day 3, P2 peak amplitudes in channels C4 and O1 were significantly lower (*p* < 0.01 and *p* < 0.05, respectively) in mafedine-treated than in untreated rats, approaching presumably healthy control levels (Figure 5C).

**Figure 6:**
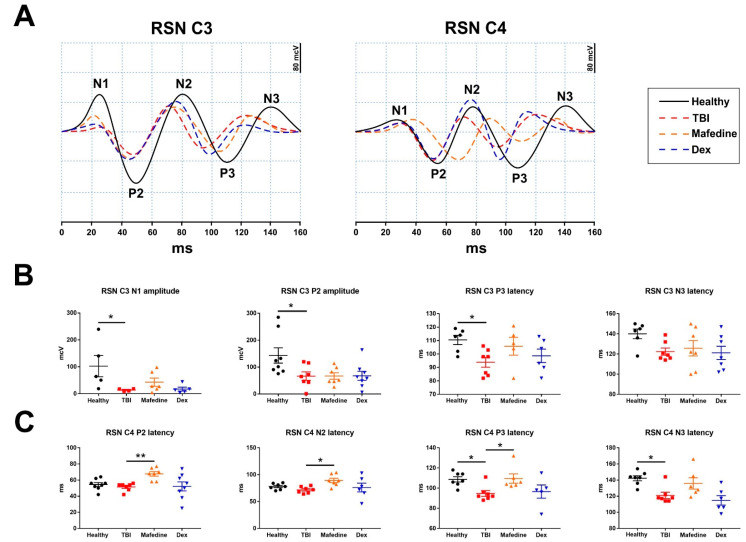
(**A**) averaged SSEP curves in channels C3 and C4 under right sciatic nerve (RSN) stimulation; (**B**) SSEP curve parameters in channel C3 under RSN stimulation. (**C**) SSEP curve parameters in channel C3 under right sciatic nerve stimulation. Dex—dexmedetomidine; * *p* < 0.05, ** *p* <0.01 vs. TBI.

Similarly, notable changes were observed in the primary-motor (channels C3 and C4) cortical responses in TBI rats at post-surgery day 7 (Figure 6A). TBI produced a significant (*p* < 0.05) decrease in the early components (N1 and P2) of SSEP induced by *n. sciaticus* stimulation in the motor cortex of the injured hemisphere (Figure 6B). Significantly (*p* < 0.05) lower latencies of the late component P3, and somewhat lower N3 peak latencies were also seen in TBI animals (Figure 6B). Similar changes were observed in the late component latencies in channel C4 (ipsilateral responses) (Figure 6C). Neither of the drugs affected the SSEP curve parameters registered in channel C3 under sciatic nerve stimulation. However, mafedine-treated rats exhibited significantly higher latencies of early (P2 and N2) and late (P3 and N3) responses in channel C4, compared with untreated animals (*p* < 0.01, *p* < 0.05) (Figure 6C).

## 4. Discussion

We have previously reported that TBI causes persistent alterations in rat brain electrical activity, which can be observed at post-TBI days 3 and 7 [6,7,8,9]. The key features of the ECoG signal in the injured animals included decreased θ-, α-, and β-wave amplitudes and indices, along with an increase in slow δ-wave activity [7]. Those changes were accompanied by weakened inter- and intrahemispheric functional connectivity as indicated by the decreased Ccr values [8]. It should be noted that the listed changes were detected in remote regions of the cortex as well as within the injured area.

Another prominent feature of the brain’s electrical activity in the injured animals was represented by the alterations in cortical responses to photic and somatosensory stimulation. In particular, brain-injured rats were found to have higher P2 VEP peak amplitudes in remote cortical areas than healthy rats at post-TBI day 3, and lower amplitudes at post-TBI day 7 [9]. Brain-injured animals also exhibited a decrease in late (N2, P2, N3) as well as early (N1, P2) cortical responses to somatosensory stimulation, had longer early-response latencies, and shorter late-response latencies [6].

We have obtained data that confirm our previous results and suggest an overall similarity in the alterations in the brain’s electrical activity observed in rat models and in human TBI patients [24,25,26]. On the one hand, this provides evidence that the controlled cortical impact model of TBI produces electrophysiological impairments similar to those seen in clinical practice. On the other hand, our findings validate the use of the presented protocol for brain electrical activity analysis to assess functional disturbances in brain-injured rats.

In a previous study employing the same animal model, mafedine administration to rats at 2.5 mg∙kg^−1^ at 1 h after TBI induction and daily for the next 6 days increased the overall locomotion activity and improved the function of contralateral fore- and hindlimbs. Mafedine-treated rats also exhibited reduced brain damage and inflammation in the injured area. Notably, mafedine had no negative impact on the behavioral characteristics of the experimental animals as indicated by the Elevated plus maze test [15].

In the present study, the beneficial impact of mafedine on the course of TBI in rats was reflected in the increase in Ccr values for channel pairs FP2-C4 and O1-O2 at post-TBI day 7. Mean θ-, α-, and β-wave amplitudes in mafedine-treated animals also tended strongly to increase towards healthy control levels. These improvements in the electrical activity of the were not observed until post-TBI day 7 and did not affect the regions of the brain which had not been directly injured. This might indicate that, despite mafedine being able to reduce brain damage and inhibit inflammation in the injured area [15], it fails to restore the full functional capacity of the surviving neurons by post-TBI day 7. The improvement in the front and hind limb motor function and the increase in total locomotion produced by mafedine [13] could likely result from a compensatory reaction of the uninjured regions of the brain. Such reactions can involve the formation of new neural connections bypassing the injured area, which has been observed in several rodent TBI models [27]. At the same time, the increase in the overall mean of the signal amplitudes and some of the wave amplitudes could be attributed to the mild psychostimulant action of mafedine, previously reported in a zebrafish model [28]. However, this does not necessarily disprove mafedine’s protective action, as some central nervous system stimulants have been found effective for posttraumatic asthenia [29].

Mafedine can positively impact cortical responses to photic and somatosensory stimulation. Administered as described above, it decreased the P2 VEP peak amplitudes in remote cortical regions, nearly restoring them to healthy control levels. This effect was only seen at post-TBI day 3; at day 7, P2 peak amplitudes in mafedine-treated rats no longer differed from those in untreated rats. Nonetheless, this observation may indicate that some of the beneficial effects of mafedine are manifest during the acute phase of TBI.

Quite interestingly, mafedine prolonged the latencies of either early or late response components in the ipsilateral cortex following sciatic nerve stimulation. Ipsilateral as well as contralateral cortex activation, induced by somatosensory nerve stimulation, has been extensively described in previous studies [30,31]. However, the exact mechanisms behind this phenomenon remain unclear. According to a hypothesis, such ipsilateral responses occur most likely due to [30]: (1) the involvement of the *corpus callosum* which transmits the stimulus between the hemispheres; (2) the uncrossed ascending fibers projecting from the stimulated nerve to the ipsilateral cortex; (3) a bifurcation of the neural impulse into both hemispheres at thalamic level. Although the delay of the ipsilateral late responses can be considered a beneficial effect of mafedine, it cannot explain the improvement in the contralateral hind limb function previously described in mafedine-treated animals [15].

In a number of studies, dexmedetomidine administration at 20–25 μg∙kg^−1^ in the acute phase of TBI has been shown to inhibit neutrophil infiltration, microglial activation, decrease plasma proinflammatory cytokine (IL1β, IL6, IL8, TNF) levels, restore blood-brain barrier integrity, prevent neuronal apoptosis, and reduce neurological deficit scores in mice [18,32]. A similar anti-inflammatory profile has been established for this drug in TBI patients [32]. These data suggest that dexmedetomidine could provide neuroprotection in brain trauma as well as ischemia [14].

In our experiment, dexmedetomidine at 20–25 μg∙kg^−1^ did not improve the electrical activity of the brain in the injured animals at post-TBI days 3 or 7 when administered as described above. This can be explained by the fact that, in most studies, dexmedetomidine was neuroprotective when administered immediately after TBI induction [18,32], following experimental protocols that suggest immediate administration after reperfusion in ischemic stroke models [33]. In one study mentioned above [32], dexmedetomidine was given to patients upon admission to the hospital, although no data were presented on the neurological deficit scores and long-term TBI outcomes. Importantly, plasma proinflammatory cytokine levels do not always correlate with the degree of neurological deficit [34]. This might suggest that although dexmedetomidine could still inhibit inflammation in the injured area, it was not sufficient enough to promote functional recovery.

Despite the relatively high amount of data acquired, our study has several significant limitations. Firstly, the dosage and administration regimen for dexmedetomidine were chosen based solely on literature data, with no preliminary experimental validation. As a result, in view of the absence of positive effects of dexmedetomidine, we cannot compare its neuroprotective profile with that of mafedine and determine whether the effects of the latter are due to the activation of alpha-2 adrenergic receptors. Secondly, we did not confirm the neuroprotective effects of mafedine using biochemical, histological, or other methods, and only used our previous results for comparison. Finally, despite the fact that long-term functional outcomes are largely determined in the acute and subacute phases of TBI, having more time points for data collection would have made the study more robust.

The possible applicability of neurophysiological techniques to evaluate neuroprotective drug candidates in in vivo studies is a relevant question for current biomedical research. Solcoseryl (deproteinated calf blood dialysate) administration to brain-injured rats during the post-traumatic period resulted in improved EEG patterns, paralleled by restored vertical and horizontal activity in the Open field test [35]. In another study, an anticonvulsant agent lacosamide normalized the delta strength of EEG simultaneously with a decrease in oxidative stress and apoptosis in traumatized rats [36]. In a rat bilateral-carotid-artery occlusion model of cerebral ischemia, pretreatment with vinpocetine and melatonin positively affected EEG parameters in surviving animals, whose number was increased to 80.0% vs. 34.8% in untreated controls [37]. Thus, experimental evidence supports the use of neurophysiological methods in neuropharmacological studies.

## 5. Conclusions

(1) TBI causes persistent alterations in brain electrical activity which can be detected using ECoG, VEP, and SSEP acquisition and analysis at post-TBI days 3 and 7;

(2) Mafedine administration at 2.5 mg∙kg^−1^ at 1 h after TBI induction and daily for the next 6 days restores inter- and intrahemispheric functional connectivity in cortical regions rostral and lateral to the injury site. Mafedine also improves the function of the sensory system, as indicated by VEP and SSEP data;

(3) Dexmedetomidine administration at 25 μg∙kg^−1^ in the same regimen does not affect the brain’s electrical activity in brain-injured animals.

## Figures and Tables

**Table 1:** Experimental schedule.

TBI Day	Post-TBI Days							
	1	2	3	4	5	6	7	
Morning: electrode implantation +TBI induction ↓ 1 h post-TBI: drug administration	12:00–13:00: drug administration	12:00–13:00: drug administration	16:00–19:00 ECoG acquisition ↓ drug administration	12:00–13:00: drug administration	12:00–13:00: drug administration	12:00–13:00: drug administration	16:00–19:00 ECoG acquisition ↓ VEP acquisition ↓ SSEP acquisition	

ECoG—electrocorticogram, VEP—visual evoked potential, SSEP—somatosensory evoked potential.

**Table 2:** Mean corticographic signal amplitudes (μV) in channels FP1, FP2, C3, C4, O1, and O2 at post-TBI days 3 and 7.

Channel	Day 3				Day 7			
	Healthy	TBI	Mafedine	Dex	Healthy	TBI	Mafedine	Dex
FP1	51.63 ± 1.67 **	32.38 ± 3.44	33.88 ± 2.27	25.63 ± 2.23	51.50 ± 1.41 *	38.29 ± 4.30	44.00 ± 2.69	35.63 ± 3.53
FP2	51.63 ± 1.24 **	37.75 ± 2.18	37.75 ± 2.81	33.38 ± 1.95	51.25 ± 1.11 *	41.57 ± 30	48.14 ± 2.62	42.50 ± 2.83
C3	69.38 ± 2.65 **	42.00 ± 2.66	41.63 ± 2.16	37.13 ± 3.74	65.63 ± 2.46 **	48.14 ± 3.40	52.43 ± 2.03	45.75 ± 3.76
C4	74.50 ± 3.85 **	48.50 ± 4.040	43.13 ± 3.99	44.88 ± 3.9	69.88 ± 2.56 *	52.00 ± 4.87	58.33 ± 3.57	52.38 ± 5.18
O1	92.63 ± 4.63 **	49.25 ± 4.46	58.38 ± 4.58	50.5 ± 6.04	86.13 ± 3.74 **	53.57 ± 4.05	70.71 ± 4.72	56.38 ± 7.66
O2	89.63 ± 3.43 **	50.75 ± 5.84	61.88 ± 3.41	56.25 ± 4.97	80.50 ± 3.81 *	54.86 ± 6.57	71.86 ± 5.76	63.00 ± 6.51

Dex dexmedetomidine; * *p* < 0.05, ** *p* < 0.01 vs. TBI.

## Data Availability

The main data are available with the corresponding author.
